# Supplementary material for: In Vitro Anti-Orthohantavirus Activity of the High-and Low-Molecular-Weight Fractions of Fucoidan from the Brown Alga Fucus evanescens
Source: Mar Drugs. 2021 Oct 15;19(10):577. doi: 10.3390/md19100577 (PMC8538225; doi:10.3390/md19100577)
Supplement: Supplementary file 1 [file marinedrugs-19-00577-s001.zip › marinedrugs-1394391-supplementary.pdf]

Supplementary material

In vitro anti-hantavirus activity of the high and low molecular weight fractions of fucoidan from the brown alga Fucus evanescens

Natalya V. Krylova[1],*, Artem S. Silchenko[2], Anastasia B. Pott[1], Svetlana P. Ermakova[2], Olga V. Iunikhina[1], A.B. Rasin[2], Galina G. Kompanets[1], Galina N. Likhatskaya[2] and Mikhail Y. Shchelkanov[1]

[1] G.P. Somov Institute of Epidemiology and Microbiology, 690087, Vladivostok, Russia; krylovanatalya@gmail.com (NVK); pott_a.b@mail.ru (ABP); olga_iun@inbox.ru (OVI); galkom1965@gmail.com (GGK); adorob@mail.ru (MYS)
[2] G.B. Elyakov Pacific Institute of Bioorganic Chemistry, 690022, Vladivostok, Russia; artem.silchencko@yandex.ru (ASS); swetlana_e@mail.ru (SPE); abrus__54@mail.ru (ABR); galin56@mail.ru (GNL)

* Correspondence: krylovanatalya@gmail.com; Tel.: +7-9084-486-423.

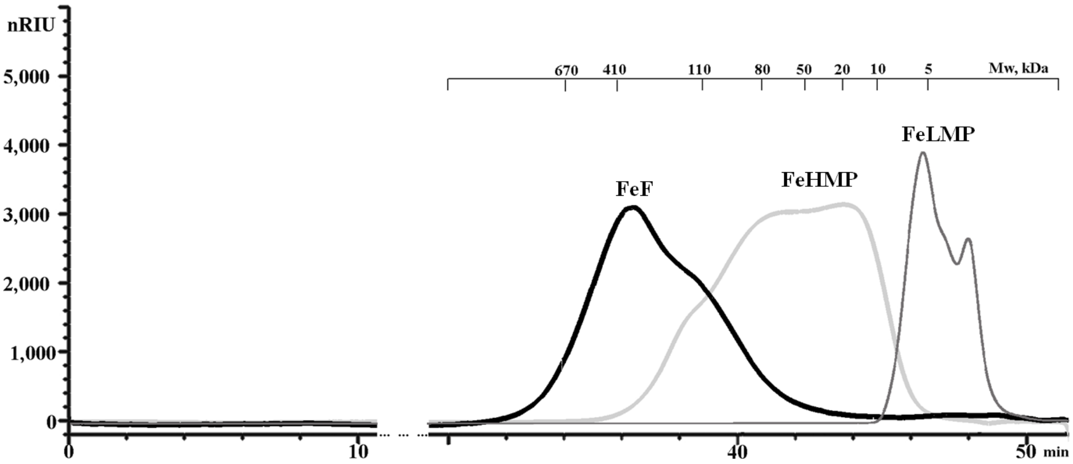

Figure S1. High-performance size-exclusion chromatography (SEC) on the series-connected columns Shodex OHpak SB-805 HQ and OHpak SB-803 HQ of the fucoidan FeF and its enzymatically prepared derivatives FeHMP and FeLMP.

Table S1. Structural characteristics of fucoidan FeF and its enzymatically prepared derivatives FeHMP and FeLMP

**Fucoidan sample**	**Molecular weight, kDa**	**-SO_3Na, %*,**	**Monosaccharide composition**		
			Fuc	Gal	Xyl
FeF	271	28	0.9	0.1	0
FeHMP	71	35	0.95	0.05	0
FeLMP	6	25	0.9	0.1	0

* % of sample weight

Table S2. Anti-AMRV activity of the fucoidans

Compounds	CC_{50}	Pretreatment of Virus		Pretreatment of Cells		Attachment		Penetration		Treatment of Infected Cells	
		IC_{50}	SI	IC_{50}	SI	IC_{50}	SI	IC_{50}	SI	IC_{50}	SI
**FeF**	> 2000	110 ± 14	18 ± 4	73 ± 10	27 ± 4	28 ± 3	72 ± 8	34 ± 4	59 ± 8	445 ± 44	4.5 ± 0.4
**FeHMP**	> 2000	97 ± 14	21 ± 4	60 ± 8	33 ± 5	18 ± 2*	113 ± 10*	30 ± 4	67 ± 7	436 ± 44	4.6 ± 0,4
**FeLMP**	> 2000	51 ± 5*	39 ± 5*	42 ± 5*	48 ± 5*	17 ± 2*	120 ± 11*	26 ± 3	75 ± 10	386 ± 43	5.2 ± 0.7
**Ribavirin**	731	NA		NA		NA		NA		20 ± 3	36 ± 5

Note: Values represent the means ± standard deviations of three or more independent experiments; FeF, native fucoidan from brown alga F. evanescens; FeHMP, high molecular weight fraction and FeLMP, low molecular weight fractions of fucoidan. Ribavirin was used as reference compound; IC_{50}, concentration that inhibited 50% of viral foci formation; SI, selectivity index (CC_{50} /IC_{50}); NA, no activity; * Significance of the differences between the parameters of modified fucoidans (FeHMP and FeLMP) compared to native fucoidan (FeF) ($p \leq 0.05$).
